# Three-Dimensional Terahertz Coded-Aperture Imaging Based on Geometric Measures

**DOI:** 10.3390/s18051582

**Published:** 2018-05-16

**Authors:** Shuo Chen, Xiaoqiang Hua, Hongqiang Wang, Chenggao Luo, Yongqiang Cheng, Bin Deng

**Affiliations:** School of Electronic Science, National University of Defense Technology, Changsha 410073, China; chenshuo13@nudt.edu.cn (S.C.); huaxiaoqiang12@nudt.edu.cn (X.H.); yqcheng@nudt.edu.cn (Y.C.); dengbin@nudt.edu.cn (B.D.)

**Keywords:** coded-aperture imaging, three-dimensional (3D), geometric measures (GMs), pulse compression

## Abstract

For synthetic aperture radars, it is difficult to achieve forward-looking and staring imaging with high resolution. Fortunately, terahertz coded-aperture imaging (TCAI), an advanced radar imaging technology, can solve this problem by producing various irradiation patterns with coded apertures. However, three-dimensional (3D) TCAI has two problems, including a heavy computational burden caused by a large-scale reference signal matrix, and poor resolving ability at low signal-to-noise ratios (SNRs). This paper proposes a 3D imaging method based on geometric measures (GMs), which can reduce the computational burden and achieve high-resolution imaging for low SNR targets. At extremely low SNRs, it is difficult to detect the range cells containing scattering information with an ordinary range profile. However, this difficulty can be overcome through GMs, which can enhance the useful signal and restrain the noise. By extracting useful data from the range profile, target information in different imaging cells can be simultaneously reconstructed. Thus, the computational complexity is distinctly reduced when the 3D image is obtained by combining reconstructed 2D imaging results. Based on the conventional TCAI (C-TCAI) model, we deduce and build a GM-based TCAI (GM-TCAI) model. Compared with C-TCAI, the experimental results demonstrate that GM-TCAI achieves a more impressive performance with regards to imaging ability and efficiency. Furthermore, GM-TCAI can be widely applied in close-range imaging fields, for instance, medical diagnosis, nondestructive detection, security screening, etc.

## 1. Introduction

Unlike synthetic aperture radars, terahertz coded-aperture imaging (TCAI) [1,2,3] can overcome the difficulties of achieving forward-looking and staring imaging with a high resolution. Referring to the imaging theory of both optical coded-aperture imaging [4,5] and radar coincidence imaging (RCI) [6,7], this imaging technology reconstructs target information with arbitrary measurement modalities, which are achieved by producing spatiotemporal independent signals with the coded aperture. Besides, terahertz waves (0.1–10 THz) have better penetrability than light and shorter wavelengths than microwaves, both of which guarantee their advantage in detecting hidden objects in security screening [8,9,10].

To develop coding devices and imaging methods for TCAI, the Defense Advanced Research Project Agency (DARPA) has proposed a project named advanced scanning technology for imaging radars (ASTIR) [11]. Recently, the Harvard Robotics Laboratory (HRL), participating in the ASTIR project, has developed a high-resolution, low-power coded aperture subreflector array (CASA) that can potentially see weapons or explosives concealed on a person, at tactically safe distances [12]. Besides, metasurfaces have also shown great promise in the flexible manipulation of terahertz and millimeter waves, which have been applied in fields of high-resolution computational imaging [13,14] and some scanning devices [15,16]. Therefore, metasurfaces are used to provide device support for TCAI.

However, due to a large number of meshed grid cells [3,17], the large-scale reference signal matrix increases computational complexity. Worse still, current TCAI algorithms lack the ability to reconstruct low signal-to-noise ratio (SNR) targets. At extremely low SNRs, the useful signal is drowned in noise, thus leading to a mismatch between the back signal and reference signal matrices. Recently, an imaging method was proposed to obtain the range profile with matched filtering [18], which is a step in pulse compression. However, when the SNR is under −10 dB, pulse compression has the additional problem of having to extract true target positions from the range profile, thus resulting in imaging failure. In simulation conditions, target positions can be extracted with knowledge of the imaging parameters. However, for practical applications, target positions are unknown.

By studying the intrinsic geometrical nature of traditional signals, information geometry provides a new way to deal with existing problems of signal processing [19]. As an important tool of information geometry, geometric measures (GMs) can transform traditional signal processing into manifold signal processing, thus improving signal-detection performance [20].

In this paper, we propose a new TCAI method based on geometric measures (GMs). The GMs’ decision maker helps extract useful range profile data, the signal quality of which is improved significantly. By constructing a covariance matrix of the range profile under TCAI architecture, the range profile data is projected into manifold space. Thus, the tough problems of 3D TCAI can be solved in the manifold with GMs. Although GMs provide a new perspective of signal processing, there are few examples of practical applications of GMs for radar imaging. We attempt to introduce GMs or information geometry into the radar imaging field. Eventually, range profile cells corresponding to different imaging planes are subdivided. Then, the three-dimensional (3D) target reconstruction is decomposed into a combination of two-dimensional (2D) images.

In this paper, Section 2 introduces the basic imaging principle and the model of conventional TCAI (C-TCAI). Then, the imaging model and procedure of GM-based TCAI (GM-TCAI) is described in detail. In Section 3, numerous experiments demonstrate the imaging ability of GM-TCAI for low SNR 3D targets. Finally, Section 4 concludes with the advantages of GM-TCAI for future applications.

## 2. Imaging Method

### 2.1. Conventional TCAI

**Figure 1:**
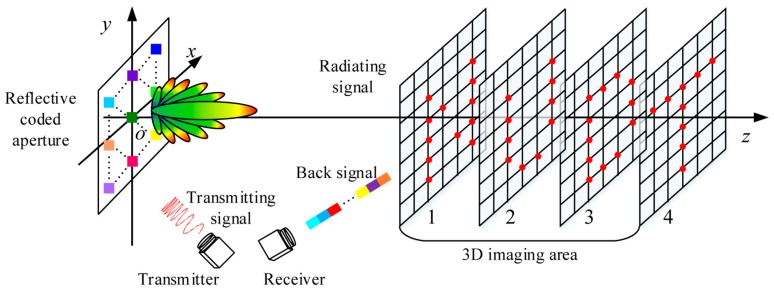
Schematic diagram of three-dimensional terahertz coded-aperture imaging (3D TCAI).

3D TCAI mainly features a transmitter, a receiver, a reflective coded aperture, and the 3D imaging area, as shown in Figure 1. The transmitter sends the transmitting signal to the reflective coded aperture, while the receiver receives the back signal with the target information. The coded aperture randomly modulates the amplitude or phase of the transmitting signal. In Figure 1, the various colors in the coded aperture denote various amplitudes or phase modulations of the transmitting signal. The 3D imaging area is subdivided into several imaging planes across various ranges. Subsequently, the divided imaging planes are further meshed into tiny grid cells, while scatters are located in the centers of the grid cells.

Firstly, the transmitter transmits a linear frequency modulation (LFM) signal, which is shown below:

(1) $$S_{t}(t)=\mathrm{rect}\left(\frac{t}{T_{p}}\right)\cdot A\cdot \exp\left[j2\pi \left(f_{c}t+\frac{1}{2}\gamma t^{2}\right)\right],$$

where $S_{t}(t)$ is the transmitting LFM signal at time t, $T_{p}$ is the pulse width, A is the amplitude, $f_{c}$ is the center frequency, γ is the chirp rate of the signal, *j* is the imaginary unit, and rect(⋅) is the rectangular window function.

For the purpose of clarity, we assume the LFM signal arriving at the coded aperture to be a plane wave. The time delay terms for each transmitting element of the coded aperture are the same, and as such, they can be set as zero. For a coded aperture containing *Q* transmitting elements, the radiating signal through the coded aperture can be expressed as:

(2) $$S_{c}(t)=\sum_{q=1}^{Q}\mathrm{rect}\left(\frac{t}{T_{p}}\right)\cdot A_{t,q}\cdot \exp\left[j2\pi \left(f_{c}t+\frac{1}{2}\gamma t^{2}\right)\right]\cdot \exp\left(j\cdot \varphi_{t,q}\right),$$

where $A_{t,q}$ and $\varphi_{t,q}$ are the random modulation terms of amplitude and phase, respectively, for the *q*th transmitting element at time *t*.

Secondly, the radiating signal illustrates the 3D imaging area, which contains K grid cells. For high-resolution imaging, the radiation field of the 3D imaging area is spatiotemporally independent. Reflected by the 3D target, the back signal arriving at the receiver is written as:

(3) $$S_{r}(t)=\sum_{k=1}^{K}\sum_{q=1}^{Q}\mathrm{rect}\left(\frac{t-t_{q,k}}{T_{p}}\right)\cdot A_{t,q}\cdot \beta_{k}\cdot \exp\left[j2\pi \left(f_{c}\left(t-t_{q,k}\right)+\frac{1}{2}\gamma {\left(t-t_{q,k}\right)}^{2}\right)\right]\cdot \exp\left(j\cdot \varphi_{t,q}\right),$$

where $\beta_{k}$ is the scattering coefficient corresponding to the *k*th grid cell, $t_{q,k}$ is the total time delay after passing though the *q*th transmitting element, the *k*th grid cell, and the receiver.

Based on the time discretion of Equation (3), the conventional mathematical model of TCAI is deduced as:

(4) $$\begin{array}{l} Sr=S\cdot \beta \\ \left[\begin{array}{c} Sr\left(t_{1}\right)\\ Sr\left(t_{2}\right)\\ \ldots \\ Sr\left(t_{N}\right) \end{array}\right]=\left[\begin{array}{cccc} S\left(t_{1},1\right) & S\left(t_{1},2\right) & \ldots & S\left(t_{1},K\right)\\ S\left(t_{2},1\right) & S\left(t_{2},2\right) & \ldots & S\left(t_{2},K\right)\\ \ldots & \ldots & \ldots & \ldots \\ S\left(t_{N},1\right) & S\left(t_{N},2\right) & \ldots & S\left(t_{N},K\right) \end{array}\right]\cdot \left[\begin{array}{c} \beta_{1}\\ \beta_{2}\\ \ldots \\ \beta_{K} \end{array}\right] \end{array},$$

where $Sr=\left(S_{r}\left(t_{n}\right)\right),n=1,\cdots N$, $S=\left(S\left(t_{n},k\right)\right),k=1,\cdots K,n=1,\cdots ,N$, and $\beta =\left(\beta_{k}\right),k=1,\cdots K$ are the back signal vector, reference signal matrix, and scattering-coefficient vector, respectively. *N* and *K* are the amount of sampling time and number of grid cells, respectively. The array element of S is as follows:

(5) $$S\left(t_{n},k\right)=\sum_{q=1}^{Q}\mathrm{rect}\left(\frac{t}{T_{p}}\right)\cdot A_{t,q}\cdot \exp\left[j2\pi \left(f_{c}\left(t-t_{q,k}\right)+\frac{1}{2}\gamma {\left(t-t_{q,k}\right)}^{2}\right)\right]\cdot \exp\left(j\cdot \varphi_{t,q}\right).$$

Based on the concept of solving linear equations, it is difficult to solve Equation (4) when there is a mismatch between the receiving signal vector, Sr, and the reference-signal matrix, S. Unfortunately, the reference signal matrix is deduced from Equation (5) under ideal conditions, while the real back signal is received with a low SNR.

### 2.2. GM-Based TCAI

**Figure 2:**
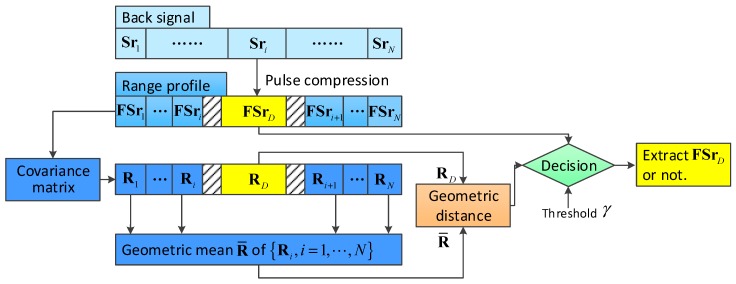
Range profile extraction based on geometric measures (GMs).

To solve the low SNR problem for TCAI, we deduced a new imaging model based on GMs, which is shown in Figure 2. The back signal matrix, $SR=\left[Sr_{1},\cdots ,Sr_{i},\cdots ,Sr_{N}\right]$, in Figure 2 is composed of *M* back signal pulses. $Sr_{i},\;n=1,2,\cdots ,N$ is a vector described as ${\left(Sr_{i1},Sr_{i2},\cdots ,Sr_{iM}\right)}^{T}$, where ${\left[\cdot \right]}^{T}$ denotes the transposition of the vector or matrix. Firstly, the range profile matrix, $FSR=\left[FSr_{1},\cdots ,FSr_{i},\cdots ,FSr_{N}\right]$, was obtained by pulse compression of the back signal matrix, SR. Even with a range profile, the target-containing range cells were still unable to be detected at extremely low SNRs. Therefore, we took advantage of GMs to extract range cells which included scattering information.

#### 2.2.1. Pulse Compression through the Dechirping Technique

3D TCAI has two problems, including a heavy computational burden caused by a large-scale reference signal matrix, and poor resolving ability at low SNRs. To solve these problems, this paper transformed the back signal from the time domain into the frequency domain with pulse compression, which is a useful method to reduce computational complexity and enhance the SNR.

For the pulse compression, we defined a reference signal, the time delay of which was set as zero. The reference signal was written as:

(6) $$S_{ref}(t)=\mathrm{rect}\left(\frac{t}{T_{p}}\right)\cdot \exp\left[j2\pi \left(f_{c}t+\frac{1}{2}\gamma t^{2}\right)\right].$$

By mixing the back signal and the reference signal, the dechirping signal was deduced as:

(7) $$\begin{array}{cc} Sr_{if}(t) & =Sr(t)\cdot S_{ref}^{*}(t)\\ & =\sum_{k=1}^{K}\sum_{q=1}^{Q}\mathrm{rect}\left(\frac{t-t_{q,k}}{T_{p}}\right)\cdot A_{t,q}\cdot \beta_{k}\cdot \exp\left[j2\pi \left(-\gamma t_{q,k}t-f_{c}t_{q,k}+\frac{1}{2}\gamma {\left(t_{q,k}\right)}^{2}\right)\right]\cdot \exp\left(j\cdot \varphi_{t,q}\right) \end{array}.$$

After Fourier transformation, the range profile could be formulated as below:

(8) $$\begin{array}{cc} FSr_{if}(f) & =F\left[Sr_{if}(t)\right]\\ & =\sum_{k=1}^{K}\sum_{q=1}^{Q}\mathrm{sinc}\left(T_{p}\left(f+\gamma t_{q,k}\right)\right)\cdot A_{q}\cdot \beta_{k}\cdot \exp\left\{j2\pi \left(-\gamma t_{q,k}f-f_{c}t_{q,k}+\frac{1}{2}\gamma {\left(t_{q,k}\right)}^{2}\right)\right\}\cdot \exp\left(j\cdot \varphi_{t,q}\right) \end{array},$$

where f is the frequency variable, F(⋅) is the Fourier transform, and $\mathrm{sinc}\left(u\right)=\frac{\sin\left(\pi u\right)}{\pi u}$ is the impulse function.

The back signal matrix, SR, was compressed row by row via Equations (7) and (8), and it was finally transformed into a range profile matrix, FSR, each column of which denoted a range cell.

#### 2.2.2. Signal Extraction by GMs

**Figure 3:**
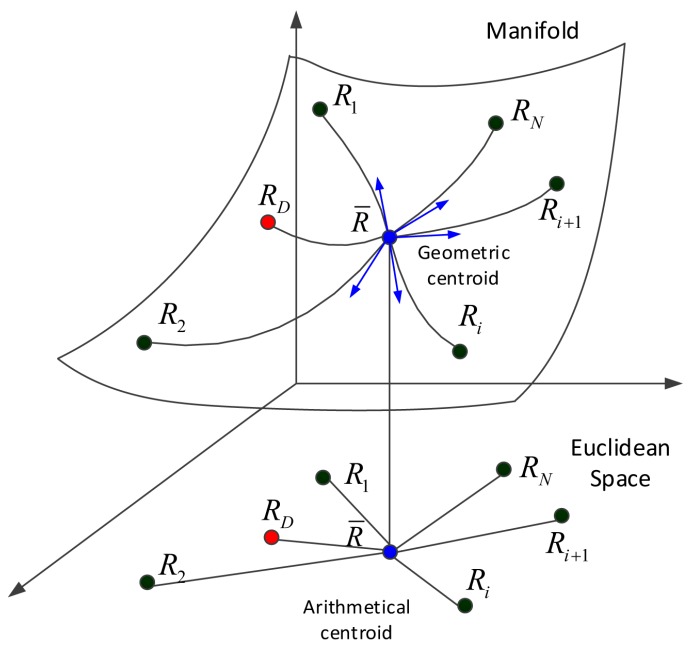
Geometrical interpretation of traditional distances in Euclidean space, and geometric divergences in manifold space, where R represents the traditional distance or geometric divergence of each element.

The range profile should have presented spike pulses at target positions. However, when the SNR was under −10 dB, it was difficult to recognize the target positions from the range profile, and thus, this resulted in imaging failure. Therefore, this paper tried to get the right positions of spike pulses by using GMs to learn the intrinsic nature of the range profile. Therefore, the following section describes the use of GMs to detect target positions, which are the foundation of successful imaging. Firstly, Figure 3 is provided to illustrate the interpretation of traditional distances into Euclidean space, and geometric divergences into manifold space. The difference between two elements was calculated as a function of their distance in Euclidean space. The detection of GMs in the manifold domain was highly improved by utilizing intrinsic divergences of the required covariance matrices. To achieve this, the Kullback–Leibler divergence (KLD) [19,20] was adopted to extract useful range profile data.

- Problem Description

This step transformed the existing problems of TCAI into a theoretical framework of information geometry. The range profile extraction could be formulated as a hypothesis problem, which was described as:

(9) $$\left\{ \begin{array}{l} {ℋ}_{0}:FSr\left(n\right)=w\left(n\right),n=1,2,\cdots ,N\\ {ℋ}_{1}:FSr\left(n\right)=s\left(n\right)+w\left(n\right),n=1,2,\cdots ,N \end{array}\right.,$$

where s(n) denotes the useful signal containing target information when FSr(n) satisfies the hypothesis ${ℋ}_{1}$, and w(n) is the correlated Gaussian noise disturbing FSr(n).

As a column of the FSR matrix, $FSr_{i}$ could be derived from either of the hypotheses described in Equation (9). To classify $FSr_{i}$, the covariance matrix, $R_{i}$, of the range profile, $FSr_{i}$, was defined by:

(10) $$R_{i}=E\left[\left(FSr_{i}\right){\left(FSr_{i}\right)}^{H}\right]=\left[\begin{array}{cccc} r_{0} & {\bar{r}}_{1} & \cdots & {\bar{r}}_{M-1}\\ r_{1} & r_{0} & \ddots & \vdots \\ \vdots & \ddots & \ddots & {\bar{r}}_{1}\\ r_{M-1} & \cdots & r_{1} & r_{0} \end{array}\right],$$

where $r_{k}=E\left[FSr_{i,m}{\bar{FSr}}_{i,m+k}\right]$ is defined as the correlation coefficient, $\bar{FSr}$ describes the complex conjugate of FSr, ${\left[\cdot \right]}^{H}$ denotes the conjugate transposition of matrix, and $R_{i}$ is a Toeplitz Hermitian positive-definite (HPD) matrix [21,22], with $R_{i}^{H}=R_{i}$. As a closed, self-dual convex cone, the HPD matrix manifold provided a higher-rank symmetric space [23]. The correlation coefficient of FSr was given by its sample mean, which was written as:

(11) $${\hat{r}}_{k}=\frac{1}{M}\sum_{m=0}^{M-1-\left|k\right|}FSr_{i,m}{\bar{FSr}}_{i,m+k},\;\left|k\right|\leq M-1.$$

The range profile data in each cell was remodeled by Equation (10), while the covariance matrix represented the target or noise information. In this way, the range profile data, $FSr_{i}=\left\{FSr_{i1},FSr_{i2},\cdots ,FSr_{i,M}\right\}$, was projected into manifold space with *M* dimensions.

B. Geometry Solution

The information of each range cell was described by its related $R_{i}$, which was an HPD matrix estimated by the range profile data, $FSr_{i}$, according to Equation (10). To decide the range cell type, we measured the difference between the detected HPD matrix, $R_{D}$, and the mean matrix, $\bar{R}$, using KLD.

The KLD-based mean [20], $\bar{R}$, of a set of HPD matrices, $\left\{R_{1},R_{2},\cdots ,R_{N}\right\}$, was calculated by

(12) $$\bar{R}=\arg\min\sum_{i=1}^{N}D^{2}\left(R||R_{i}\right)={\left(\frac{1}{N}\sum_{i=1}^{N}R_{i}^{-1}\right)}^{-1},$$

where D(⋅) denotes the KLD calculation.

Finally, the decision was made by comparing the distance between $R_{D}$ and $\bar{R}$ with an adaptive detection threshold, γ.

According to ref. [20], the KLD between two HPD matrices, $R_{1}$ and $R_{2}$, is formulated as:

(13) $$D\left(R_{1},R_{2}\right)=\mathrm{tr}\left(R_{2}^{-1}R_{1}-I\right)-\log_{10}\det\left(R_{2}^{-1}R_{1}\right),$$

where tr(⋅) denotes the matrix trace, while det(⋅) describes the matrix determinant.

Then, the KLD-based decision maker is performed with:

(14) $$D\left(R_{D},\bar{R}\right)\overset{{ℋ}_{0}}{\underset{{ℋ}_{1}}{≶}}\gamma ,$$

where γ is the threshold between noise and target range cells. When $D\left(R_{D},\bar{R}\right)>\gamma$, the range cell contains target information, and its corresponding $FSr_{D}$ will be indexed and extracted.

C. Range Profile Extraction

Unlike $FSR=\left[FSr_{1},\cdots ,FSr_{i},\cdots ,FSr_{N}\right]$, the range profile matrix, FSR, can be described in another form, which is shown as:

(15) $$FSR=\left[{\left(FSr^{1}\right)}^{T},\cdots ,{\left(FSr^{i}\right)}^{T},\cdots ,{\left(FSr^{M}\right)}^{T}\right],$$

where $FSr^{i}={\left(FSr^{i1},FSr^{i2},\cdots ,FSr^{iN}\;\right)}^{T}$ denotes the row element of FSR.

According to Equation (8), the range profile $FSr^{i}$ presents a spike pulse at $f=-\gamma t_{q,k}$, where $t_{q,k}=r_{q,k}/c$, and $r_{q,k}$ is the distance delay corresponding to $t_{q,k}$. Therefore, the scattering information within the same range gathers in the same spike pulse. The 3D imaging area in Figure 1 has four imaging planes across various ranges. As each imaging plane was located in several range cells adjacent to one another, the range profile vector, $FSr^{i}$, showed four spike pulses. On one hand, the four spike pulses included all the information of the 3D target. On the other hand, each spike pulse only contained the target information within its imaging plane. As described in Section 2.2.2, the corresponding spike pulses of $FSr^{i}$ were indexed and extracted using GMs. Subsequently, the target in each imaging plane was reconstructed one by one, and then synthesized together to obtain the entire 3D target.

**Figure 4:**
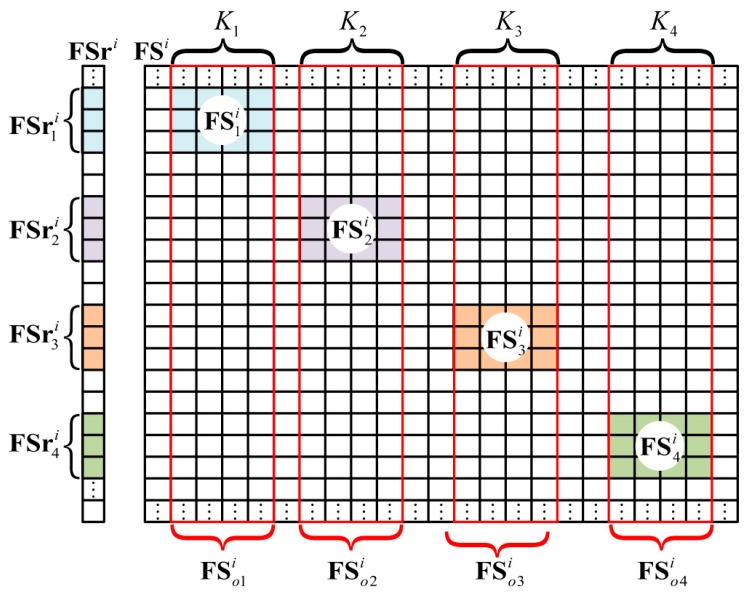
Extraction of the range profile vector, and conformation of the range profile reference signal matrix.

Using GMs, different spike pulses in $FSr^{i}$ were extracted separately. For example, in Figure 1, $FSr^{i}$ could be subdivided into $FSr_{1}^{i}$, $FSr_{2}^{i}$, $FSr_{3}^{i}$, and $FSr_{4}^{i}$, as shown in Figure 4. Moreover, $r_{1}$, $r_{2}$, $r_{3}$, and $r_{4}$ were indexed as corresponding row positions of $FSr_{1}^{i}$, $FSr_{2}^{i}$, $FSr_{3}^{i}$, and $FSr_{4}^{i}$ in $FSr^{i}$.

#### 2.2.3. Conformation of the Reference Signal Matrix

As shown in Figure 4, $K_{1}$, $K_{2}$, $K_{3}$, and $K_{4}$ were the numbers of the grid cells in the four imaging planes. $FS^{i}$ was the range profile reference signal matrix related to $FSr^{i}$, while $FS_{1}^{i}$, $FS_{2}^{i}$, $FS_{3}^{i}$, and $FS_{4}^{i}$ were the range profile reference signal matrices corresponding to $FSr_{1}^{i}$, $FSr_{2}^{i}$, $FSr_{3}^{i}$, and $FSr_{4}^{i}$, respectively. Moreover, $FS_{1}^{i}$, $FS_{2}^{i}$, $FS_{3}^{i}$, and $FS_{4}^{i}$ were partly extracted from $FS_{o1}^{i}$, $FS_{o2}^{i}$, $FS_{o3}^{i}$, and $FS_{o4}^{i}$, which were later introduced. Apparently, only $FS_{1}^{i}$, $FS_{2}^{i}$, $FS_{3}^{i}$, and $FS_{4}^{i}$ needed to be constructed, instead of the whole matrix, $FS^{i}$.

Firstly, the time-domain reference signal matrices $S_{1}^{i}$, $S_{2}^{i}$, $S_{3}^{i}$, and $S_{4}^{i}$ were deduced from Equations (4) and (5).

Similar to Equations (7) and (8), we dechirped and transformed each column of $S_{1}^{i}$, $S_{2}^{i}$, $S_{3}^{i}$, and $S_{4}^{i}$ into the frequency domain. For example, S(t,k), the reference signal in the *k*th column, could be processed with:

(16) $$S_{if}\left(t,k\right)=S\left(t,k\right)\cdot S_{ref}^{*}(t),$$

(17) $$FS_{if}(f,k)=F\left[S_{if}\left(t,k\right)\right].$$

Through pulse compression, we obtained the original range profile reference signal matrices, $FS_{o1}^{i}$, $FS_{o2}^{i}$, $FS_{o3}^{i}$, and $FS_{o4}^{i}$.

As shown in Figure 4, the row numbers of $FS_{o1}^{i}$, $FS_{o2}^{i}$, $FS_{o3}^{i}$, and $FS_{o4}^{i}$ were the same as that of $FSr^{i}$. As described in the section describing range profile extraction, $r_{1}$, $r_{2}$, $r_{3}$, and $r_{4}$ were the row position tags of $FSr_{1}^{i}$, $FSr_{2}^{i}$, $FSr_{3}^{i}$, and $FSr_{4}^{i}$. As such, we could use $r_{1}$, $r_{2}$, $r_{3}$, and $r_{4}$ to extract corresponding rows of $FS_{o1}^{i}$, $FS_{o2}^{i}$, $FS_{o3}^{i}$, and $FS_{o4}^{i}$, and finally get the required range profile reference signal matrices, $FS_{1}^{i}$, $FS_{2}^{i}$, $FS_{3}^{i}$, and $FS_{4}^{i}$.

#### 2.2.4. Imaging Model Based on GMs

With *M* pulses denoted in Figure 2, we could combine all range profile vectors and reference signal matrices. For example, with an imaging plane named *x*, the synthesized range profile vector and reference signal matrix could be written as:

(18) $$FSr_{x}^{\mathrm{GM}}={\left[{\left(FSr_{x}^{1}\right)}^{T},\cdots ,{\left(FSr_{x}^{i}\right)}^{T},\cdots ,{\left(FSr_{x}^{M}\right)}^{T}\right]}^{T},$$

(19) $$FS_{x}^{\mathrm{GM}}={\left[{\left(FS_{x}^{1}\right)}^{T},\cdots ,{\left(FS_{x}^{i}\right)}^{T},\cdots ,{\left(FS_{x}^{M}\right)}^{T}\right]}^{T}.$$

Then, the mathematical model based on a decision made using geometric measures could be formulated as:

(20) $$FSr_{x}^{\mathrm{GM}}=FS_{x}^{\mathrm{GM}}\cdot \beta_{x},$$

where $\beta_{x}$ is the scattering coefficient vector of imaging plane *x*. Based on this model, each imaging plane in Figure 1 could be reconstructed in parallel to reduce the computational burden. Finally, the imaging results of all imaging planes were combined to reconstruct the 3D target.

The high-resolution image was then obtained through GM-TCAI. Table 1 shows the whole imaging procedure below.

## 3. Experimental Results

In this section, the range profile cells containing scattering information were indexed by GM divergence, using KLD. Each cluster cell corresponded to one imaging plane at a fixed range. Through the useful compressed sensing (CS) algorithm, total variation (TV) regularization can recover both sparse and extended targets [24]. By incorporating TV regularization, both sparse and extended targets were tested to compare C-TCAI and GM-TCAI at extremely low SNRs. We adopted the relative imaging error (RIE) and probability of successful imaging (PSI) [25] to estimate the performance of GM-TCAI.

Firstly, the RIE was defined as:

(21) $$\mathrm{MSE}≜20\log_{10}\left({\left\|\hat{\beta }-\beta \right\|}_{2}/{\left\|\beta \right\|}_{2}\right),$$

where $\hat{\beta }$ and β are the estimated and true targets, respectively.

Secondly, the PSI was defined as:

(22) $$\mathrm{PSI}≜\min{\left(\hat{\beta }\right)}_{\Lambda }/\max{\left(\bar{\hat{\beta }}\right)}_{\Lambda },$$

where Λ denotes the correct positions containing targets, ${\left(\hat{\beta }\right)}_{\Lambda }$ carries the same values as $\hat{\beta }$ at Λ, and ${\left(\bar{\hat{\beta }}\right)}_{\Lambda }$ takes zeroes at Λ, and the same values as $\hat{\beta }$ at the other positions. Herein, the presence of successful imaging was proportional to the value of the PSI.

The basic parameters in the experiments are shown in Table 2. The 3D imaging area included four imaging scenes, which denoted the imaging planes across four different ranges. By adding 0 or π, the coded aperture randomly modulated the phase of the transmitting signal. The computer performing the experiments was equipped with an i5-6200U processor, and 8 GB of memory.

### 3.1. Range Profile Extraction Based on GMs

**Figure 5:**
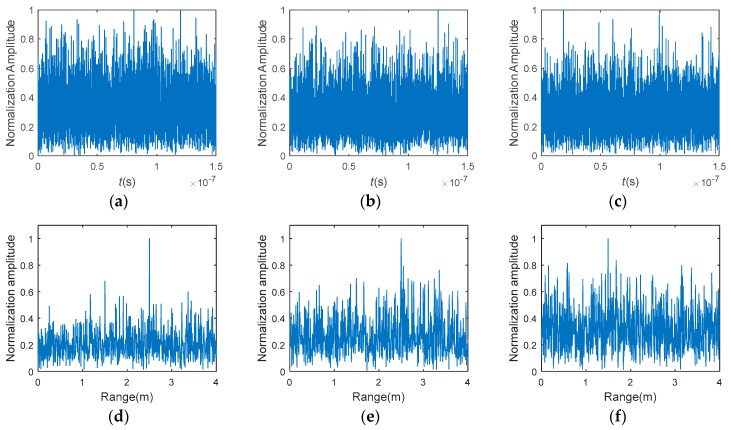

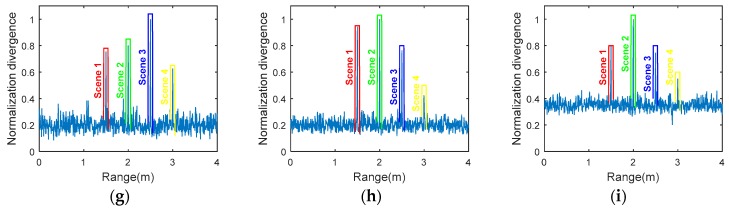
(**a**–**c**) The back signals at −15 dB, −20 dB, and −25 dB, respectively; (**d**–**f**) the range profiles with pulse compression at −15 dB, −20 dB, and −25 dB, respectively; (**g**–**i**) the range profiles processed with GMs at −15 dB, −20 dB, and −25 dB, respectively.

According to Equation (4), C-TCAI reconstructed the target through the original back signals, which are shown in Figure 5a–c. To obtain the range profile, the back signal vector was processed with pulse compression, which is described in detail in Section 2.2.1. As the 3D imaging area contained four scenes or imaging planes, the range profiles in Figure 5d–f should have presented four spike pulses around 1.5 m, 2 m, 2.5 m, and 3 m. However, the SNR was too low to detect the target position. Referring to Equation (10), each range cell corresponded to an HPD matrix, $R_{i}$. To detect the true target position, the GM divergences between each HPD matrix, $R_{i}$, and the mean HPD matrix, $\bar{R}$, were calculated. As shown in Figure 5g–i, the spike pulses corresponding to scenes 1, 2, 3, and 4 were easy to judge, and were marked with red, green, blue, and yellow rectangular boxes, respectively. With differing range information located in each spike pulse, it was easy to divide and extract the range profiles of each scene. Using the processed signal, target reconstructions of the four scenes could be performed simultaneously at low SNRs.

### 3.2. Imaging Results for Sparse Targets

The sparse targets of “N”, “U”, “D”, and “T” shapes were distributed in scenes 1, 2, 3, and 4, respectively. NUDT is the abbreviation of the National University of Defense Technology. To simulate the original back signals for TCAI, various radiation patterns were convolved with the scattering coefficients in the 3D imaging area. For the sparse targets, ten pulses were adopted to obtain the back signal matrix, SR, which was compressed to construct the range profile matrix, FSR. Through the GMs’ decision maker, the imaging data was extracted from the range profile. C-TCAI and GM-TCAI were based on Equations (4) and (20), respectively. Referring to the four scenes in the 3D imaging area, the size of the reference signal matrix for C-TCAI was 3600 × 3600, while the size for GM-TCAI was 900 × 900. Therefore, the computational complexity of C-TCAI was much larger than that of GM-TCAI. Using the imaging method of TV regularization, Table 3 presents the time consumption of C-TCAI and GM-TCAI for the sparse targets. Apparently, the imaging time of GM-TCAI was much shorter than that of C-TCAI.

**Figure 6:**
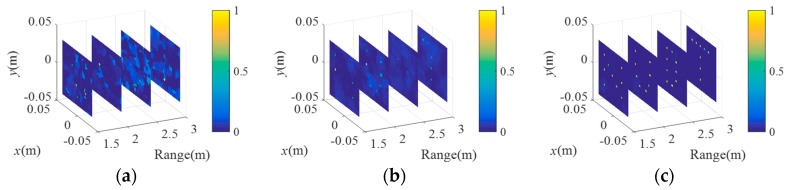

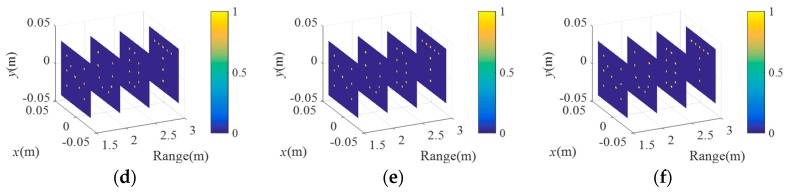
Comparison of imaging results for C-TCAI and GM-TCAI for sparse targets at various SNRs. (**a**–**c**) Imaging results for C-TCAI at −25 dB, −15 dB, and −5 dB; (**d**–**f**) imaging results for GM-TCAI at −25 dB, −15 dB, and −5 dB.

Figure 6 gives comparisons of the imaging results of C-TCAI and GM-TCAI at various SNRs. Figure 6a–c describes the results of C-TCAI at −25 dB, −15 dB, and −5 dB, while Figure 6d–f shows the results of GM-TCAI at −25 dB, −15 dB, and −5 dB. When the SNRs were −25 dB and −15 dB, C-TCAI resolved nothing of the targets, as shown in Figure 6a,b. Even at −5 dB, C-TCAI could only reconstruct a blurred image with some unclear scatters. As shown in Figure 6d–f, GM-TCAI could reconstruct all target points with correct positions and scattering information. Therefore, the performance of GM-TCAI was deemed impressive at extremely low SNRs.

**Figure 7:**
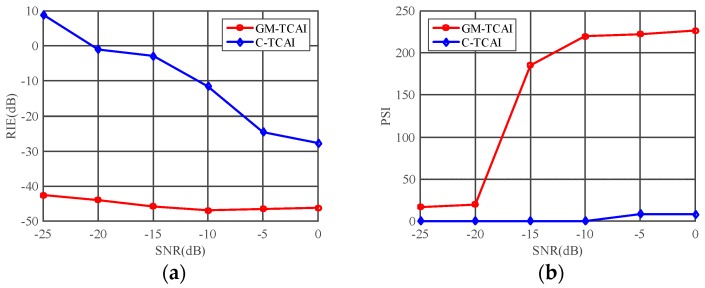
Imaging evaluations of conventional TCAI (C-TCAI) and GM-based TCAI (GM-TCAI) using: (**a**) the relative imaging error (RIE); and (**b**) the probability of successful imaging (PSI), at various SNRs for sparse targets.

The RIE and PSI were used to quantitatively evaluate the imaging results. Figure 7a presents the RIE comparisons of C-TCAI and GM-TCAI. The RIEs of GM-TCAI were always lower than those of C-TCAI. Especially at lower SNRs, the difference in RIEs between GM-TCAI and C-TCAI was much larger. As shown in Figure 7b, the PSIs of GM-TCAI were always higher than one, where PSI>1 denotes successful imaging. Therefore, GM-TCAI could always achieve successful imaging, regardless of the SNR. However, the PSIs of C-TCAI were always too small to guarantee successful imaging. Considering the runtime comparison, GM-TCAI could achieve efficient and high-resolution imaging for sparse targets.

### 3.3. Imaging Results for Extended Targets

The extended targets with “N”, “U”, “D”, and “T” shapes were located in scenes 1, 2, 3, and 4, respectively. As the extended targets were more complex than the sparse targets, fifty pulses were used to obtain the back signal matrix, SR, and construct the range profile matrix, FSR. Using the imaging method of TV regularization, Table 4 presents the time consumption of C-TCAI and GM-TCAI for the extended targets. Obviously, GM-TCAI could save more time than C-TCAI. Compared with Table 3, extended target reconstruction needed more time than sparse target reconstruction.

**Figure 8:**
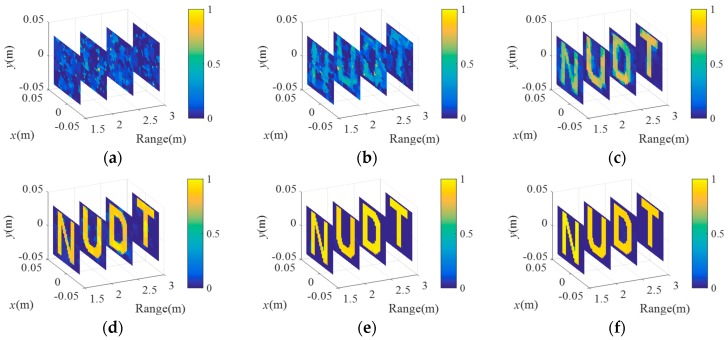
Comparison of imaging results for C-TCAI and GM-TCAI for extended targets at various SNRs. (**a**–**c**) Imaging results for C-TCAI at −25 dB, −15 dB, and −5 dB; (**d**–**f**) imaging results for GM-TCAI at −25 dB, −15 dB, and −5 dB.

For the extended targets, Figure 8 presents comparisons of the imaging results of C-TCAI and GM-TCAI at various SNRs. When the SNRs were −25 dB and −15 dB, it was difficult to distinguish the target in the imaging results from C-TCAI, as shown in Figure 8a,b. At −5 dB, C-TCAI could only reconstruct the blurred contour of the target, as shown in Figure 8c. Despite some background noise in Figure 8d, GM-TCAI could clearly reconstruct the useful target information. As shown in Figure 8e,f, GM-TCAI performed better and better with increasing SNRs. Thus, GM-TCAI could also reconstruct the extended target with excellent performance at low SNRs.

**Figure 9:**
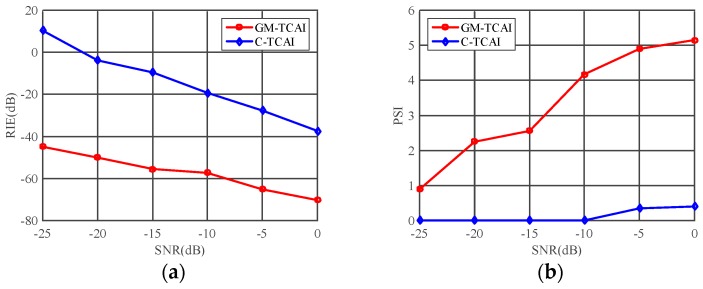
Imaging evaluations of C-TCAI and GM-TCAI using: (**a**) the relative imaging error (RIE); and (**b**) the probability of successful imaging (PSI), at various SNRs for extended targets.

Using the RIE and PSI, Figure 9 presents the quantitative comparison of C-TCAI and GM-TCAI. Similar to those in Figure 7a, the RIEs of GM-TCAI in Figure 9a were always lower than those of C-TCAI. As shown in Figure 9b, the PSIs of GM-TCAI could guarantee successful imaging, while the PSIs of C-TCAI denoted difficulty in obtaining successful imaging. Therefore, GM-TCAI was an effective imaging method for both sparse and extended 3D targets.

## 4. Conclusions

This paper proposed a 3D imaging method based on GMs to reduce computational burden and achieve high-resolution imaging for low SNR targets. The GMs’ decision maker extracted the useful range profile data, which was categorized into various imaging planes. The range profile reference signal matrices were then constructed corresponding to extracted range profile data for each imaging plane. Based on C-TCAI, we deduced a GM-TCAI model. Finally, numerical experimental results demonstrated that our imaging method achieved efficient imaging with less computational burden, and high-resolution imaging for both sparse and extended targets at low SNRs. Thus, GM-TCAI holds promising practicability for close-range imaging fields, such as security checks, medical diagnosis, nondestructive detection, etc. Although GMs have opened a new gate for radar imaging, few theories of information geometry have been applied to practical radar imaging. Through the imaging solution presented in this paper, we hope that more radar imaging problems can be solved with information geometry theories by other researchers.

## Figures and Tables

**Table 1:** Imaging procedure of geometric measure-based terahertz coded-aperture imaging (GM-TCAI).

Input	Back signal matrix, $SR=\left[Sr_{1},Sr_{2},\cdots ,Sr_{N}\right]$, with *M* back signal vectors.
Step 1	Obtain the range profile matrix, $FSR=\left[FSr_{1},\cdots ,FSr_{i},\cdots ,FSr_{N}\right]$, via Equations (7) and (8).
Step 2	Obtain the HPD matrices, $R_{1},R_{2},\cdots ,R_{N}$, from $FSr_{1},\cdots ,FSr_{i},\cdots ,FSr_{N}$ via Equation (10).
Step 3	Calculate the mean KLD, $\bar{R}$, of the HPD matrices, $R_{1},R_{2},\cdots ,R_{N}$, via Equation (14).
Step 4	*for I* = 1:*N*, compare the divergence between $R_{i}$ and $\bar{R}$ referring to Equation (12), and extract the range profile vectors, $FSr_{1}^{i}$, $FSr_{2}^{i}$, $FSr_{3}^{i}$, and $FSr_{4}^{i}$, via Equation (14). *end* *Return*: (1) The row position tags, $r_{1}$, $r_{2}$, $r_{3}$, and $r_{4}$, of the imaging planes containing targets. (2) Combination of the extracted range profile vectors, $FSr_{1}^{\mathrm{GM}}$, $FSr_{2}^{\mathrm{GM}}$, $FSr_{3}^{\mathrm{GM}}$, and $FSr_{4}^{\mathrm{GM}}$, via Equation (18).
Step 5	Construct the range profile reference signal matrices, $FS_{1}^{\mathrm{GM}}$, $FS_{2}^{\mathrm{GM}}$, $FS_{3}^{\mathrm{GM}}$, and $FS_{4}^{\mathrm{GM}}$, corresponding to $FSr_{1}^{\mathrm{GM}}$, $FSr_{2}^{\mathrm{GM}}$, $FSr_{3}^{\mathrm{GM}}$, and $FSr_{4}^{\mathrm{GM}}$ via Equation (19) and the procedure detailed in Section 2.2.3.
Step 6	Reconstruct ${\overset{⌢}{\beta }}_{1}$, ${\overset{⌢}{\beta }}_{2}$, ${\overset{⌢}{\beta }}_{3}$, and ${\overset{⌢}{\beta }}_{4}$ from different imaging planes, according to Equation (20).
Return	Obtain the initial 3D imaging result, $\overset{⌢}{\beta }$, through a combination of ${\overset{⌢}{\beta }}_{1}$, ${\overset{⌢}{\beta }}_{2}$, ${\overset{⌢}{\beta }}_{3}$, and ${\overset{⌢}{\beta }}_{4}$.

**Table 2:** Primary parameters used in the experiments.

Parameter	Value
Center frequency (*f_c_*)	340 GHz
Bandwidth (B)	20 GHz
Pulse Width (*T_p_*)	100 ns
Size of the coded aperture	0.5 m × 0.5 m
Number of coded-aperture array elements	25 × 25
Sampling frequency (*f*_s_)	25 GHz
Range of Scene 1	1.5 m
Range of Scene 2	2 m
Range of Scene 3	2.5 m
Range of Scene 4	3 m
Size of the grid cell	0.0025 m × 0.0025 m
Number of grid cells in each scene	30 × 30
GM divergence	Kullback–Leibler divergence (KLD)

**Table 3:** Runtime for sparse target.

	Conventional TCAI	GM-TCAI
Runtime	41.4877 s	1.1040 s

**Table 4:** Runtime for extended target.

	C-TCAI	GM-TCAI
Runtime	51.1916 s	14.6427 s
